# Case Report: Splanchnic Vein Thrombosis as a Complication of Necrotizing Acute Pancreatitis in a Pediatric Patient

**DOI:** 10.3389/fsurg.2022.747671

**Published:** 2022-04-01

**Authors:** Mauricio Figueroa-Sánchez, Carlos M. Nuño-Guzmán, M. Carmen Álvarez-López, Mariana Ordónez-Cárdenas, Leidy J. Montaño-Rodríguez

**Affiliations:** ^1^Department of Radiology and Imaging, Hospital Civil de Guadalajara Fray Antonio Alcalde, Guadalajara, Mexico; ^2^Centro Universitario de Ciencias de la Salud, Universidad de Guadalajara, Guadalajara, Mexico; ^3^Department of General Surgery, Hospital Civil de Guadalajara Fray Antonio Alcalde, Guadalajara, Mexico; ^4^Department of Pediatric Gastroenterology, Hospital Civil de Guadalajara Fray Antonio Alcalde, Guadalajara, Mexico

**Keywords:** splanchnic vein thrombosis, portosplenomesenteric venous thrombosis, acute pancreatitis, pediatric patients, vascular complications

## Abstract

Splanchnic vein thrombosis is an unusual manifestation of venous thromboembolism and includes portal vein thrombosis, mesenteric veins thrombosis, splenic vein thrombosis, and the Budd-Chiari syndrome. The most common risk factors include hematologic and autoimmune disorders, hormonal therapy, liver cirrhosis, solid abdominal cancer, recent abdominal surgery, and abdominal infections or inflammatory conditions, such as pancreatitis. Splanchnic vein thrombosis in acute pancreatitis is most commonly associated with the severe form of the disease and pancreatic necrosis. This report describes a case of splanchnic vein thrombosis as a complication of necrotizing acute pancreatitis in a pediatric patient. Splanchnic vein thrombosis was incidentally detected on contrast-enhanced computed tomography to assess the pancreas. There was no evidence of prior risk factors for the thrombotic condition. The patient was treated with anticoagulation and showed complete resolution after recovery from necrotizing acute pancreatitis, at a 16-month follow-up. The complication of necrotizing acute pancreatitis with splanchnic vein thrombosis in pediatric age is a rare presentation.

## Introduction

Splanchnic vein thrombosis (SVT) is an unusual manifestation of venous thromboembolism (VTE), and includes portal vein thrombosis (PVT), mesenteric veins thrombosis (MVT), splenic vein thrombosis (SplVT), and the Budd-Chiari syndrome (BCS). Approximately two thirds of SVT cases occur in male patients. The peak incidences of BCS, PVT, and MVT occur at 45–50, 54–61, and 70–79 years, respectively (1).

The most common risk factors associated with PVT and MVT include local precipitating factors such as solid abdominal cancer, liver cirrhosis, intraabdominal inflammatory conditions and surgery, while BCS is most commonly related with systemic risk factors such as hematological disorders, autoimmune disease and hormonal therapy (2). SVT may manifest as a complication of acute pancreatitis (AP). The etiopathogenic mechanism of SVT in AP involves a direct inflammatory process, and is most commonly associated with severe AP. The identification of pain due to SVT may be difficult in patients with AP, but its incidence has been reported between 1 and 24% (3).

Herein we report a case of SVT in the presence of necrotizing AP in a pediatric patient. The SVT was incidentally detected by a contrast-enhanced computed tomography (CECT) to evaluate the pancreas.

## Case Presentation

A 14-year-old Mexican female patient, presented to the emergency room of our hospital with a two-day history of epigastric pain, and gastrobiliary content vomiting. At physical examination, the patient appeared tachycardic and mildly dehydrated. The epigastric and mesogastric tenderness were elicited. Her past history was unremarkable and there were no previous similar clinical events. The patient had an unremarkable family medical history. At laboratory examination, amylase level was 561 U/L (normal range 40–140 U/L). Lipase level was 2,556 U/L (normal range 0–160 U/L). The patient was diagnosed with idiopathic AP as there was no history of drug or alcohol intake, and blood and image studies for other etiologies were normal. The patient and her mother agreed to be treated under no oral intake, intravenous (IV) fluid administration and analgesic medication.

**Figure 1:**
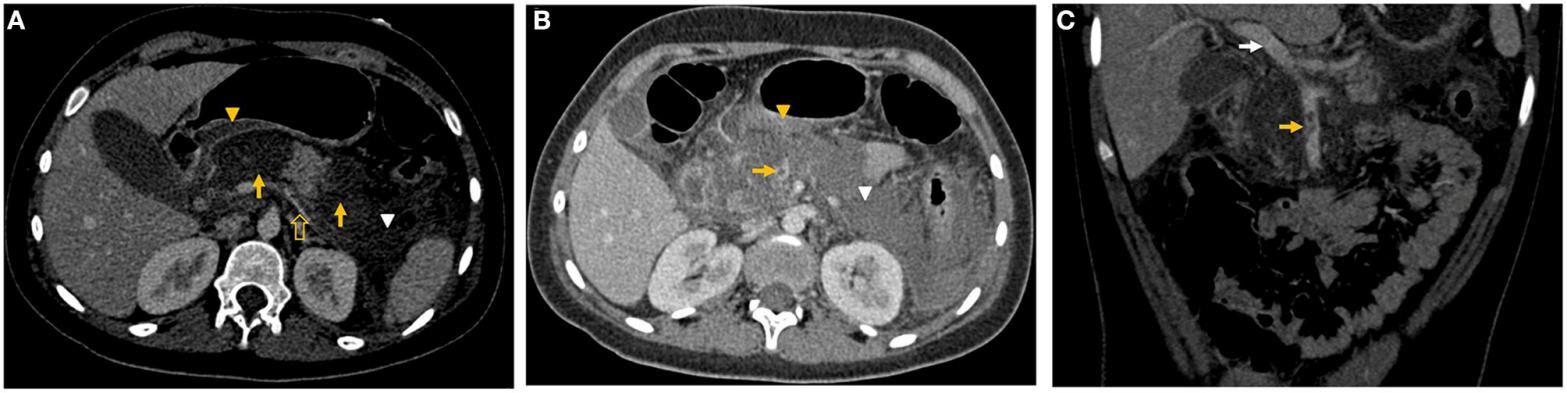
**(A)** Axial CECT view showed 80% parenchymal necrosis, affecting head, neck and tail (yellow arrows), APFC adjacent to pancreatic head (yellow arrowhead) and in left anterior pararenal space (white arrowhead), and nonocclusive SplVT (yellow hollow arrow). **(B)** Nonocclusive SMVT (yellow arrow), APFC adjacent to pancreatic head (yellow arrowhead) and in left anterior pararenal space (white arrowhead) are shown. **(C)** Coronal CECT view of non-occlusive SMVT (yellow arrow), and normal portal vein (white arrow).

At day 3 of admission the patient continued with severe abdominal pain. A CECT was performed and showed diffuse enlargement of the pancreas and 80% parenchymal necrosis. Acute peripancreatic fluid collections (APFC) adjacent to pancreatic head and in left anterior pararenal space were observed (Balthazar E) (Figure 1A). Non-occlusive (SplVT) (Figure 1A) and superior mesenteric vein thrombosis (SMVT) (Figure 1B) were also visualized, with occlusion of 50 and 30%, respectively, but their confluence was patent (Figure 1C). There were no image signs of portal hypertension. The patient and her mother agreed on a nasojejunal (NJ) tube and enteral nutrition through the NJ tube. The patient continued on IV fluid restitution and enteral nutrition through the NJ tube was instituted. The patient and her mother were informed about the benefits and risks of anticoagulation, which they accepted. A 3-month period of anticoagulation with low-molecular-weight heparin (LMWH), subcutaneous enoxaparin (1 mg/kg/dose q12h) was initiated.

At day 21, a second CECT was performed due to clinical deterioration, and no evidence of SMVT or SplVT was found (not shown). An image-guided aspiration of the fluid collections was performed, and after a positive Gram stain, antimicrobial therapy with meropenem was initiated. Anticoagulation was withdrawn 12 h before and 12 h after image-guided aspiration. At day 51, the patient was discharged after improved clinical condition, under enoxaparin at same dosage and monthly follow-up. The patient and her mother accepted to continue under out-patient treatment and follow-up.

## Outcome And Follow-Up

**Figure 2:**
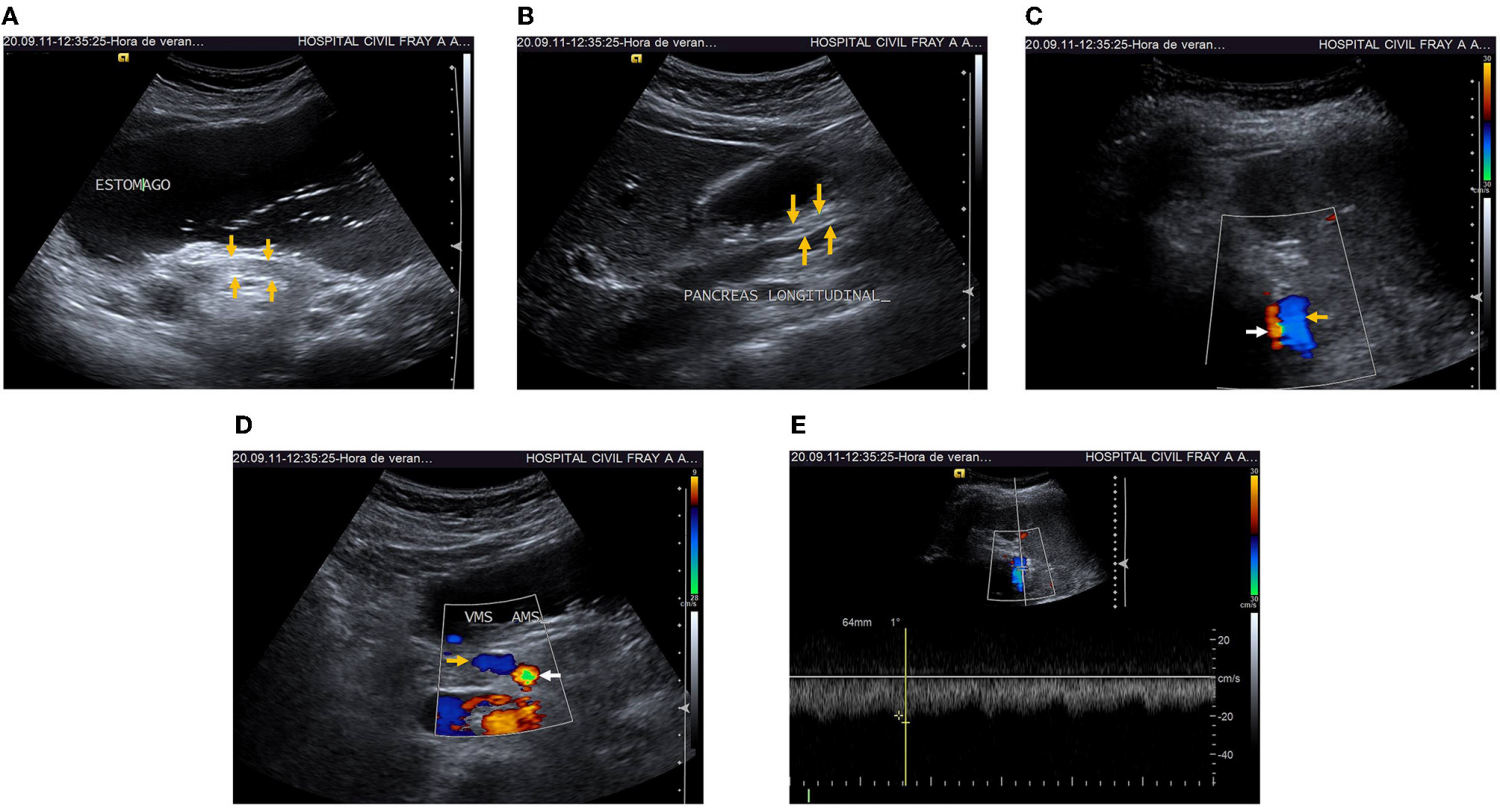
**(A)** 16-month follow-up transverse US showed thin pancreatic head and tail (arrows) and absence of fluid collections. **(B)** Longitudinal US showed thin pancreatic head and tail (arrows), absence of fluid collections. **(C)** Doppler-Ultrasonography (DUS) at 16-month follow-up showed resolution of SplVT (yellow arrow) and normal splenic artery (white arrow). **(D)** DUS showed complete resolution of SMVT (yellow arrow), and normal superior mesenteric artery (white arrow). **(E)** Pulsed-DUS, normal velocity in splenic vein (23.5 cm/s).

After monthly follow-up by the treating clinicians, anticoagulation was discontinued after completing the three-month course, with complete adherence, adequate tolerance and no secondary effects. At 16-month of follow-up by the treating clinicians, the patients continued in good clinical condition, and symptom-free. An US was performed. A thin pancreatic head and tail and absence of fluid collections were depicted (Figures 2A,B). A Doppler US (DUS) showed absence of SMVT and SplVT (Figures 2C,D). Pulsed-DUS demonstrated a normal velocity in SplV (23.5 cm/s) (Figure 2E). There were no signs of portal hypertension. Table 1 shows the timeline with relevant data from the episode of care.

**Table 1:** The timeline with relevant data from the episode of care.

**Time**	**Event**
Day 0	Admission.
3 days later	Severe abdominal pain was complained. A CECT was performed and showed diffuse pancreatic enlargement and 80% parenchymal necrosis. Non-occlusive SplVT and SMVT were depicted. A three month period of enoxaparin was initiated.
21 days later	A second CECT was performed due to clinical deterioration. There was no evidence of SMVT of SplVT. An image-guided aspiration sample of fluid collections resulted in positive Gram stain, and antimicrobial therapy with meropenem was initiated.
51 days later	The patient was discharged after clinical improvement under monthly follow-up.
16 months later	At follow-up, the patient continued in good clinical condition. DUS showed sustained permeability of SVT and SplV. Pulsed-DUS depicted a normal velocity in SplV.

The patient's written informed consent for this publication was not required by the Ethics Committee of our hospital. The patient's identity was protected.

## Discussion

SVT is a rare presentation of VTE. The incidence of VTE has been reported in the range of 70–270 cases per 100,000 persons-years, but SVT incidence is at least 25 times lower (1). Myeloproliferative neoplasms and thrombophilic disorders are the leading causes of SVT. Systemic risk factors which include hematologic and autoimmune disorders and hormonal therapy are the most common risk factors associated with BCS. Local factors for SVT include liver cirrhosis, solid abdominal cancer, recent abdominal surgery, and abdominal infections or inflammatory conditions, such as pancreatitis, cholecystitis, appendicitis, diverticulitis, liver abscess and inflammatory bowel diseases (2, 4).

SVT is a well recognized local vascular complication of AP. It may manifest as SplVT, PVT and SMVT, either separately or in combination. It is often detected incidentally on imaging performed for evaluation of symptoms and/or complications of AP. However, SVT may present with hepatic decompensation due to PVT, small bowel ischemia due to SMVT, and upper gastrointestinal bleeding from gastroesophageal varices due to SplVT and/or PVT (5). SVT is precipitated by venous intimal injury, which may be caused by inflammation and cellular infiltration, venous compression and stasis by the edematous pancreas, peripancreatic collections or a pseudocyst, leading to venous stasis, platelet and fibrin-rich thrombin deposition caused by systemic activation of hemostasis, and coagulation cascade initiation by exposure of disrupted pancreatic tissue factor to blood. A systemic hypercoagulable or prothrombotic state has been demonstrated in experimental studies (3). A systematic analysis reported that the pooled prevalence of SVT in AP was 16.6%. The pooled prevalence of PVT, SplVT and MVT in AP were 8.0, 10.4 and 2.6%, respectively (6). SVT has been most commonly associated with severe AP, particularly with pancreatic necrosis (3, 7–9). SVT has been reported in up to 50% of patients with necrotizing AP (10). SplVT thrombosis may present in up to 45% of such patients as a result of its proximity to the dorsal pancreatic surface (9, 11, 12).

Clinical manifestations of SVT may be difficult to differentiate from those of other abdominal conditions. The most common symptom is abdominal pain. Patients may also present gastrointestinal bleeding from gastroesophageal varices secondary to portal hypertension. Frequent manifestations also include ascites, hepatomegaly and splenomegaly. Approximately 30% of patients remain asymptomatic. Detection of incidental SVT has increased due to the extensive use of CT imaging (13). Clinical presentation of SVT in AP depends on the time of development and circulatory obliteration. It may difficult to differentiate pain due to thrombosis from pain due to AP. SVT may also manifest by an abdominal mass which corresponds to splenomegaly, bleeding secondary to portal hypertension, or cytopenias due to hypersplenism, but the risk of such complications appears to be low (3, 11, 12). A systematic review and meta-analysis by Butler et al. found that 51.9% of pancreatitis-induced SVT patients presented splenomegaly. Variceal formation was observed in 53% of patients, but only 12.3% presented gastrointestinal bleeding (14). SVT is frequently an incidental finding on image studies practiced to assess the severity of AP (7).

Prompt diagnosis of SVT is important to avoid complications. D-dimer is a well-known biomarker for deep vein thrombosis and pulmonary thromboembolism, but has shown limited utility in SVT (1, 4, 13). When there is suspicion of SVT, DUS is the first line diagnostic image modality. DUS shows a sensitivity of 89%-93% and specificity of 92%-99% for PVT. DUS may also allow detection of ascites and splenomegaly. However, it may be limited by the body habitus of the patient and operator expertise (1, 4, 15).

The current standards for diagnosis of SVT are CT angiography and MR angiography. These image modalities can show the PVT, SMVT and SplVT with an unenhancing area of lower attenuation, which corresponds to the thrombus. CT can also evaluate the bowel wall and mesentery for signs of ischemia. Sensitivity and specificity for CT in diagnosis of MVT are 91–95 and 94–100%, respectively (1, 4). MR angiography is indicated in patients with contraindications to CT angiography (1, 13). Conventional angiography is currently reserved for patients with high clinical suspicion of MVT and who are potential candidates for endovascular therapeutic procedures (13).

In patients with AP, CECT allows evaluation for the presence of pancreatic edema, necrosis and peripancreatic fluid collections. Splanchnic evaluation in portal venous phase includes assessment for vascular patency, thrombosis and narrowing. Evaluation also requires assessment for collaterals, ascites, mesenteric edema, splenic infarcts and bowel ischemia (10, 12). In a cohort study of AP patients, Easler et al. reported an overall SVT prevalence of 14 and 18% among patients who had a CECT. The risk of SVT in patients with necrotizing AP was about 50%. Considering this risk estimate of 50% and a prevalence of necrotizing AP of 5–10%, the prevalence of SVT in AP in community or non-tertiary hospitals would be 2–5% (12). Follow-up by repeat DUS has been performed to inspect the status of fluid and necrotic collections, and the course of SVT for progression or resolution of the thrombus (8).

The routine indication of anticoagulation in SVT secondary to AP has been a matter of debate. Anticoagulation is associated with improved survival, lower risk of recurrence, and improved recanalization, but also with gastrointestinal bleeding risk. Anticoagulation is recommended for symptomatic SVT patients and no evidence of active bleeding. The decision to institute anticoagulation in patients with incidentally detected and asymptomatic SVT should be individualized, balancing the risk factors for progression and recurrence, and the risk of bleeding. Anticoagulation should be maintained for at least 3 months in all SVT patients, as was the case in our patient, or indefinitely if underlying prothrombotic factors persist (2, 16–19). Pancreatic and peripancreatic inflammation plays a predominant role in SVT development. SplV is the most commonly affected vessel and has shown a high rate of spontaneous recanalization, and patients that may benefit from anticoagulation are those with SplVT extending into the PV and/or SMV, bowel ischemia, liver decompensation and underlying thrombophilia disorders (5). A recently published practice guidance does not recommend antithrombotic therapy for patients with pancreatitis and isolated SplVT, but considering the uncertainty about who will develop spontaneous recanalization, a close monitoring is required. The practice guidance recommends antithrombotic therapy for patients with MVT extension and with clinical manifestations of bowel ischemia (20). Unfortunately, there is no specific recommendation for SVT and asymptomatic MVT, as in our case.

The routine use of anticoagulation in SVT secondary to AP may increase the risk of bleeding and limit interventions such as pancreatic collection drainage. A recent meta-analysis demonstrated that there was no significant difference in thrombus resolution, varices or collaterals formation, or mortality between patients under anticoagulation and those without it. Nevertheless, anticoagulation was associated with a significant higher rate of bleeding. This meta-analysis includes a limited number of observational studies with small sample sizes and no solid conclusions can be made (21).

SVT in children has been associated with both hereditary and acquired factors. Risk factors for SVT in pediatric patients include conditions that cause direct vessel injury, umbilical vein catheterization, rare portal vein congenital anomalies, and systemic causes such as neonatal sepsis, abdominal sepsis, dehydration, multiple exchange transfusions, and congenital and acquired hypercoagulable states (22, 23).

In recent decades, there has been an increase in AP incidence in children. Most of AP diagnostic and management guidelines are based on adult patient studies, but the etiology in children is often drugs, infections, trauma or anatomic variants, whereas in adult AP is most commonly due to gallstones or alcohol intake. Clinical manifestations and course of disease are often different than in adults (24, 25). Reports of an increasing incidence of AP in children have been possible due to increased awareness and advances in diagnostic approach (26, 27). Diagnosis of AP in children is based on the INSPPIRE criteria, which were adapted after the Atlanta criteria in adults (28, 29).

We report a case of necrotizing AP in a pediatric patient which was complicated with SVT. The etiology was undetermined. The association of AP and SVT in children is an extremely rare condition, with an incidence of <1%. Venous thrombosis was been observed in recurrent AP and chronic pancreatitis (30). di Francesco et al. reported three pediatric cases of association of PVT and pancreatitis. There was evidence to suggest the preexistence of PVT prior to pancreatitis, and that the pancreatic disease was secondary to cavernomatous transformation of the regional venous system. The three patients showed resolution of pancreatitis and its complications after portal system decompressive surgery (31). In contrast, in our case there was no evidence of risk factors for SVT prior to the onset of AP, and there was complete resolution of SVT after recovery from necrotizing AP. Our patient showed complete resolution of SMVT and SplVT and no signs of portal hypertension at 16-month follow-up.

We present a case of SVT as a complication of necrotizing AP in a pediatric patient, which was incidentally detected on CECT. After resolution of acute pancreatitis and anticoagulation, there was SMVT and SplVT resolution. The complication of necrotizing AP with SVT in pediatric age is a rare presentation. The decision to institute anticoagulation in patients with incidentally detected SVT should be individualized, balancing the risk factors for progression and recurrence, and the risk of bleeding.

## Data Availability Statement

The original contributions presented in the study are included in the article/supplementary material, further inquiries can be directed to the corresponding author/s.

## Author Contributions

MF-S, MÁ-L, MO-C, and LM-R: acquisition, analysis, and interpretation of data for the work. MF-S and CN-G: drafting the work. CN-G: final approval of the version to be published. All authors contributed to the article and approved the submitted version.

## Conflict of Interest

The authors declare that the research was conducted in the absence of any commercial or financial relationships that could be construed as a potential conflict of interest.

## Publisher's Note

All claims expressed in this article are solely those of the authors and do not necessarily represent those of their affiliated organizations, or those of the publisher, the editors and the reviewers. Any product that may be evaluated in this article, or claim that may be made by its manufacturer, is not guaranteed or endorsed by the publisher.
